# Reporting is not supporting: Why mandatory supporting, not mandatory reporting, must guide university sexual misconduct policies

**DOI:** 10.1073/pnas.2116515118

**Published:** 2021-12-22

**Authors:** Kathryn J. Holland, Elizabeth Q. Hutchison, Courtney E. Ahrens, M. Gabriela Torres

**Affiliations:** ^a^Assistant Professor of Psychology and Women’s & Gender Studies, University of Nebraska–Lincoln, Lincoln, NE 68588;; ^b^Professor of History, Associate Vice President, Division for Equity and Inclusion, The University of New Mexico, Albuquerque, NM 87131;; ^c^Professor of Psychology, California State University, Long Beach, CA 90840;; ^d^Professor of Anthropology, Associate Provost for Academic Administration and Faculty Affairs, Wheaton College, Norton, MA 02766

Policies that require most or all university employees to report any sexual misconduct they learn about to a designated university official, including victims’ names, even if the victim does not want such a report to be made, have been widely implemented in institutions of higher education (1). Despite the widely held belief that these “universal” mandatory reporting policies are both necessary and effective for addressing sexual misconduct, Title IX guidance and rulemaking have never required this practice and there is little evidence that confirms the efficacy of these policies (1, 2). As the Biden administration considers changes to the 2020 Title IX regulations and state legislatures consider laws that impose broad mandatory reporting policies in higher education (e.g., Texas Senate Bill 212 passed in 2019 and California Senate Bill 493 passed in 2020), we must evaluate the impact of broad mandatory reporting policies that compel involuntary disclosure and implement evidence-based, survivor-centered ways to provide effective support to victims of sexual misconduct in higher education.

The National Academies of Science, Engineering, and Medicine (NASEM) has engaged this issue through its 2018 report *Sexual Harassment of Women: Climate, Culture, and Consequences in Academic Sciences, Engineering, and Medicine* (3), pointing to growing research on the negative effects of universal mandatory reporting. At the 2020 Public Summit, the NASEM Action Collaborative on Preventing Sexual Harassment in Higher Education convened Title IX practitioners and sexual violence researchers to further examine the consequences of universal mandatory reporting for survivors and employees, particularly when reports must include the victim’s name and when targets of sexual misconduct do not wish to report their experience to a university official. In this article, we build upon the discussions held in this panel and suggest alternative reporting policies that will better support survivors.

## Ungrounded Assumptions behind Mandatory Reporting Policies

There are several problematic assumptions embedded in universal mandatory reporting policies. First, disclosures are assumed to be de facto reports. However, there is an important difference between disclosure and reporting: Disclosure involves directly and intentionally telling someone about a personal experience, whereas reporting constitutes asking someone in authority to take official action (4). Although some survivors may disclose to university employees with the expectation that they will take official action—in which case the employee should be required to report it—others may simply be seeking support, information, and/or accommodations (5–7).

Other assumptions underlying universal mandatory reporting policies are linked to campus safety strategies that are more aspirational than reality-based. It is assumed that universal mandatory reporting will surface more cases of sexual misconduct that would otherwise go unreported, enabling institutions to identify perpetrators (especially serial perpetrators) and respond promptly and effectively. Mandatory reporting is also assumed to protect and benefit survivors, which conflates the act of reporting sexual misconduct with supporting its targets. There is little evidence to support either assumption (1). In fact, emerging evidence suggests that broad mandatory reporting policies that compel disclosures can discourage survivors from seeking help and disclosing to employees they trust (8–11). Even when reports are made, university conduct processes are only sought out in one of four of these cases, suggesting that compelled disclosures rarely lead to investigations, hearings, or sanctions (12). Moreover, policies that require all university employees to report assume that staff and faculty are not vulnerable to the sexual harassment that pervades the academic workplace, a reality that shapes employees’ willingness to report sexual misconduct they experience or witness (3, 13). In short, mandatory reporting policies may not be doing what we think they are doing. The intractability of sexual harassment in academia, and how to combat this issue, has been the sustained focus of the Action Collaborative on Preventing Sexual Harassment in Higher Education (14). If one of our goals is to implement effective policy, we must acknowledge the ungrounded assumptions in university mandatory reporting policies and examine the empirical evidence detailing the harms of overly broad mandatory reporting.

## Mandatory Reporting without Consent Harms Survivors

According to the US Department of Health and Human Services Substance Abuse and Mental Health Services Administration (SAMHSA), one of the key principles of a trauma-informed approach to working with survivors includes empowerment, voice, and choice (15). This is because regaining a sense of control is central to recovery and healing after sexual trauma (16–20). When support providers take control away, survivors report increased posttraumatic stress, depression, and anxiety (21–23). It is therefore unsurprising that survivors prefer reporting policies that grant them autonomy and control over the decision to report (7).

Mandatory reporting may also exacerbate intersectional harms experienced by marginalized students who are most vulnerable to sexual violence, including students of color, international students, and LGBTQ students (24). Universal mandatory reporting brings survivors into contact with campus offices for Title IX investigation and compliance, which can reinforce the mistrust that persons of color already have in campus safety approaches (24–26). Raced–gendered stereotypes, overpolicing, racial profiling, and personal or vicarious experiences of police brutality have led to low reporting rates by Black, Indigenous, and Latinx populations (27, 28). Thus, universal reporting mandates may be particularly harmful to and dissuade racialized persons from disclosing sexual misconduct and seeking supportive services (25, 29, 30).

University staff and faculty who are themselves targets of sexual misconduct face the above harms, as well as the professional costs (up to and including retaliation) that sexual harassment reports frequently incur (31). While tenured faculty may well fear retaliation from more powerful perpetrators of misconduct, university staff, untenured faculty (including adjuncts and lecturers), and graduate students risk their course of study or employment for reporting sexual misconduct (32, 33).

## Mandatory Reporting Policies Interfere with University Teaching and Research

Universal mandatory reporting also has negative implications for the core missions of academic institutions, including academic freedom in teaching and research. At many institutions, faculty members must report sexual misconduct that they learn about in a classroom context, such as classroom discussions and assignments (34), including assaults that occurred before the student even entered college (10, 11). Assignments that incorporate biographical writing, self-reflection, and discussion board posts all run the risk of student disclosure (35). For faculty who regularly teach about violence or trauma, mandatory reporting makes it nearly impossible to construct teaching experiences that truly facilitate learning (36, 37).

Faculty are often encouraged to include syllabi statements informing students that if they discuss personal victimization their experiences will be reported to university officials (38). Such statements can have a silencing effect on classroom discussions, and faculty may change the nature of class assignments or avoid such discussions altogether in an effort to avoid reporting requirements (36, 39). Consequently, faculty are presented with an impossible choice—continue to teach about sexual violence, knowing that you will eventually be forced to betray a student’s confidence, or simply stop teaching about sexual violence. While research on faculty behavior is sparse, anecdotal evidence suggests that mandatory reporting policies have changed the way some faculty teach, reducing the nature and amount of content, discussions, and assignments on sexual misconduct (40).

A similar dilemma is faced by faculty who conduct research on sexual misconduct. If the research design involves longitudinal research, interviews, incentives, or any other means of participant identification, researchers may be required to report their research participants’ information to university officials, often in direct violation of institutional review board (IRB) confidentiality requirements (41, 42). Empirical research is essential for improving understanding of sexual violence, identifying supportive efforts for survivors, informing empirically supported policy, and developing and testing prevention programming (3). Although some institutions may allow the Title IX coordinator to grant reporting exceptions for IRB-approved human subjects research, this practice is not guaranteed and researchers are reporting the exclusion of sexual assault survivors in their research due to their institution’s mandatory reporting policy (43).

## Recommendations: Reporting Practices That Center Support

Whatever good intentions might have originally inspired the widespread adoption of universal mandatory reporting policies, the evidence of their harm—to survivors, university employees, and the mission of higher education—demands that we reassess what it would really mean to support survivors and guarantee their access to education under Title IX. The best way to do this is to embrace a principle of mandatory supporting. Making the paradigm shift from mandatory reporting to mandatory supporting will require careful policy changes and continued employee training, but these institutional investments are no more difficult (and much more promising) than those required by the existing mandatory reporting regime. What effective supporting would require is that university employees be required to listen to and respect survivors’ intentions when they disclose, which may or may not include making a report to university officials. To fully center mandatory supporting, the Department of Education should require that colleges and universities:

• Create and/or expand confidential supportive campus services, including confidential advocacy and ombuds services, that survivors can access without contacting the Title IX office;
• Provide and require training for university employees to support survivors by responding to disclosures in a trauma-informed and racially and sexually inclusive manner, communicating the reporting options and resources that are available to them, and referring them to campus and community advocates and health providers for confidential assistance;
• Adopt a discloser-centered reporting policy that requires university employees to ask whether the survivor wants to report the incident of sexual misconduct to university officials, without attempting to encourage or discourage reporting, and make a report if the survivor gives consent;
• Ensure that, if mandatory supporting policies are implemented, they do not require supportive intervention when there is no intentional disclosure, including situations where employees learn about sexual violence:
  • at public awareness events (e.g., Take Back the Night, candlelight vigils, protests, speak-outs),
  • in social media posts (e.g., using #metoo),
  • in academic class discussions and work products (e.g., in an assignment),
  • during the hiring or admissions process (e.g., personal statements, interviews),
  • in IRB-approved human subjects research, and
  • in campus climate surveys; and
• Expand anonymous and voluntary reporting options that survivors can control. For instance, third-party reporting technologies, such as the nonprofit Callisto, enable survivors to create time-stamped records of their assault and control when a report is forwarded to university officials (e.g., if another person names the same perpetrator).

Given the empirical evidence, we argue that a mandatory supporting approach will be far more likely to fulfill the ultimate goal of Title IX and remove barriers to academic success than do current mandatory reporting policies. University mandatory reporting policies presently offer few options for survivors to control what happens to their personal information when university employees learn about experiences of sexual misconduct. Lack of consent lies at the heart of both sexual assault and universal mandatory reporting. Rather than automatically reporting sexual misconduct to university officials without survivors’ consent, a mandatory supporting approach would require employees to provide information to survivors—including their reporting options and resources for professional support—so they can make informed choices about how to proceed (44). If we want to increase reporting, we need to ensure that survivors feel safe and supported. With respect to teaching, our colleges and universities need more safe space for education and discussion about sexual misconduct, not less. Research is also necessary for developing evidence-based policies, prevention programs, and supportive services and must not be obstructed by mandatory reporting. Mandatory supporting avoids the harms to survivors and the mission of academic institutions that are caused by broad mandatory reporting policies.
